# Synergistic Effect of miR-383 and Cisplatin on Inhibition of Growth, Proliferation, and Migration of Lung Cancer Cells

**DOI:** 10.34172/aim.33450

**Published:** 2025-05-01

**Authors:** Vagef Elyaszadeh, Maryam Tohidast, Seyed Samad Hosseini, Mohammad Amini, Parinaz Marami, Behzad Baradaran, Amir Ali Mokhtarzadeh, Asiyeh Jebelli

**Affiliations:** ^1^Department of Biological Science, Faculty of Basic Science, Higher Education Institute of Rab-Rashid, Tabriz, Iran; ^2^Immunology Research Center, Tabriz University of Medical Sciences, Tabriz, Iran; ^3^Department of Cell and Molecular Biology, Faculty of Biological Sciences, Kharazmi University, Tehran, Iran

**Keywords:** Apoptosis, Cisplatin, Combination therapy, Lung cancer, miR-383

## Abstract

**Background:**

Lung cancer (LC) is a common life-threatening malignancy in humans. Cisplatin has been widely used in the treatment of various types of cancer. miR-383 is dysregulated in multiple cancers, and participates in tumorigenic processes, including apoptosis, proliferation, metastasis, and drug resistance. This study aimed to investigate the synergistic effect of miR-383 and cisplatin in LC.

**Methods:**

A549 cells were treated with cisplatin and miR-383 separately or in combination. Cell viability, apoptosis induction, stemness features, migratory capacity, and autophagy were measured by various methods. In addition, quantitative real-time PCR (qRT‐PCR) was used to evaluate the expression levels of genes involved in apoptosis, stemness, and migration.

**Results:**

The results demonstrated that miR-383 transfection in A549 cells increased their chemosensitivity to cisplatin, enhancing cisplatin-induced apoptosis (from 11.28% to 37.86%). This effect was mediated by regulating key genes such as *Bcl-2* and *Caspase-3* (*P*<0.0001). Moreover, the combination of miR-383 and cisplatin synergistically reduced cell migration and colony formation. It also downregulated metastatic and stemness-related genes, including *MMP-2* and *CD44*, respectively (*P*<0.0001).

**Conclusion:**

The findings indicate that the combination treatment of miR-383 and cisplatin suppressed cell proliferation, migration and colony formation while enhancing the sensitivity of A549 cells to chemotherapy compared to monotherapy. These results suggest that miR-383 combination therapy warrants further investigation as a potential strategy for LC treatment.

## Introduction

 Lung cancer (LC) is one of the most frequently diagnosed malignancies, accounting for approximately 2.2 million new incident cases and resulting in an estimated 1.79 million fatalities annually, making it the primary contributor to global cancer-related mortality. Overall, LC causes more deaths than colorectal, breast, prostate, and brain cancers combined. The two main types of LC are small cell lung cancer (SCLC) and non-SCLC (NSCLC). SCLC comprises about 15%, and NSCLC accounts for approximately 85% of all cases of LC.^1-3^ Despite improvements in clinical treatment and surveillance strategies, LC has a poor prognosis. Risk factors for NSCLC have been recognized, and smoking was identified as the main factor along with other genetic and environmental risk factors. Current treatment options for LC depend on the stage of cancer development, and LC patients receive certain treatments including surgery, radiotherapy, chemotherapy, and targeted therapy.^4^ Cisplatin and other platinum-based anticancer drugs have been used widely to treat cancers such as ovarian, lung, testicular, colorectal, bladder, head and neck, cervical, esophageal, and sarcoma cancers. Although cisplatin is extensively used in chemotherapy, it is associated with substantial dose-limiting adverse effects and drug resistance. Hence, cisplatin has been combined with other anticancer agents to improve its clinical utility.^5,6^ Fortunately, many attempts have been made to identify the molecular mechanisms and pathways involved in cisplatin sensitivity/resistance. Combination therapy with chemotherapy and immunotherapy has become a standard treatment for some types of cancer, with significant survival benefits and improved efficacy. Recent meta-analyses have highlighted its effectiveness, particularly in progression-free and overall survival. This approach targets multiple mechanisms, helping overcome resistance.^7^ microRNAs (miRNAs) are promising mediators of cisplatin sensitivity or resistance due to their involvement in diverse cellular processes like proliferation, differentiation, migration, and angiogenesis.^8^ miRNAs regulate gene expression via modulation of multiplex target mRNAs. Imperfect complementarity between the mRNA and miRNA leads to the blocking of translation, while perfect complementarity leads to mRNA degradation.^9^ miR-383 is located on chromosome 8p22, and it has been identified to be downregulated in multiple cancers such as hepatocellular carcinoma,^10^ embryonal carcinoma,^11^ medulloblastoma,^12^ glioma,^13^ breast cancer,^14^ ovarian cancer,^15^ colorectal cancer,^16^ and LC.^17^ Several investigations have illustrated the involvement of miR-383 in tumorigenesis, encompassing apoptosis, proliferation, invasion, metastasis, and resistance to drugs. While targets downstream like LDHA, CCND1, IGF, and VEGF have been identified as regulated by miR-383, its precise functions in tumorigenesis and the underlying mechanisms remain unclear.^18^ It has been reported that miR-383 expression is reduced in LC due to chromosome 8p22 deletion and it reduces cisplatin sensitivity. miR-383 suppresses RBM24, which activates NF-kB signaling and promotes chemoresistance. RBM24 inhibition restores chemosensitivity. Targeting miR-383/RBM24/NF-kB interactions may provide therapeutic strategies to overcome chemotherapy resistance in LC.^19^ Furthermore, it was indicated that miR-383 is significantly downregulated in the exosomes of cisplatin-resistant HeLa cells. Ectopic expression of miR-383 enhances cisplatin sensitivity in HeLa cells by targeting VEGF and inhibiting the Akt signaling pathway. These findings highlight miR-383’s role in overcoming cisplatin resistance via horizontal transfer.^20^ Targeting miRNAs, either increasing or decreasing their expression, seems to be a fascinating approach for developing novel and more beneficial personalized treatments, increasing drug effectiveness, and predicting patient response to different treatments.^21^ The present research investigated the impact of combining miRNA-383 with cisplatin on LC cells. The study assessed the combined therapy’s effects on proliferation, apoptosis, migration, colony formation, and autophagy. Additionally, quantitative real-time PCR (qRT‐PCR) was used to evaluate the expression levels of associated genes. The distinctive aspect of this study is the investigation of miR-383’s role in enhancing LC cell sensitivity to cisplatin. Based on these considerations, our hypothesis is that combination treatment of LC cells with miRNA-383 and cisplatin can enhance drug efficacy. Furthermore, this approach may be potentially used to overcome drug resistance.

## Material and Methods

###  Cell Culture

 A549 cells, derived from human type II alveolar epithelial cells, play an important role in lung metabolism and immune responses. They exhibit high proliferation, express key metabolic enzymes, activate KRAS, MAPK, and PI3K/AKT pathways, and are widely used for anticancer drug sensitivity and toxicity assays.^22^ The A549 cell line was acquired from the Pasture Institute of Iran and cultured in RPMI‐1640 medium supplemented with 10% fetal bovine serum (FBS) from Gibco, along with 100 U/mL penicillin/streptomycin mixtures. These cultured cells were then maintained in a humidified environment with 5% CO_2_ and 95% air at 37 °C.^23^ In all assays, a group of untreated cells was included as a control to account for potential confounding factors, ensuring that variations in transfection efficiency or cisplatin sensitivity are accurately assessed. All instruments used in the experiments were standard and properly calibrated to ensure measurement accuracy.

###  microRNA Transfection

 First, 1 × 10^6^ A549 cells were dissolved in an electroporation buffer and transferred to a 500 µL cuvette. Then, different concentrations of miR-383 (10, 20, and 40 pmol) were added. Subsequently, transient transfection of miRNA was achieved following the established protocol, employing the Gene Pulser Xcell electroporation system by BioRad. Cells were then seeded at a concentration of 1 × 10^4^ cells per well in 96-well plates and incubated for 48 hours. The impact of miR-383 transfection on cellular viability was evaluated using the MTT assay. The optimal dosage of miR-383, as determined from the MTT results, was chosen for the ensuing experiments. Transfection efficiency was monitored using an electroporation system with standardized parameters to ensure consistent delivery. Additionally, the optimal miR-383 concentration was selected based on MTT assay results, indirectly reflecting transfection efficiency.^24^

###  MTT Assay

 To evaluate the potential enhancement of A549 cell sensitivity to cisplatin treatment by miRNA-383, the MTT assay was conducted. First, the cells were subdivided into four groups: miR-383, cisplatin, combined miR-383/cisplatin, and the control group. To ensure consistency, the initial cell density was kept uniform across all wells (1 × 10⁴ cells per well in a 96-well plate) and incubated for 24 hours. Subsequently, they were exposed to varying concentrations of cisplatin (ranging from 1 to 100 μg/mL). Following another 24-hour incubation, to account for potential inter-assay variability, 50 μL of MTT solution (2 mg/mL in PBS) was introduced into each well and left to incubate for 4 hours. Afterward, the MTT solution was aspirated, and 200 μL of DMSO was added, followed by an additional 45-minute incubation at 37 ℃. The absorbance of the wells was measured using an ELISA reader (Sunrise, Tecan, Switzerland) at 570–630 nm.^25^ To minimize variability and ensure accurate quantification of cell viability, absorbance values were normalized to the untreated control group (set to 100% viability), and background absorbance (wells without cells) was subtracted from all measurements. Experiments were performed in triplicate to ensure reproducibility, and cell viability was expressed as a percentage of the control group.

###  RNA Extraction, cDNA Synthesis and qRT ‐ PCR

 Cellular RNA was isolated using TRIzol reagent (South Korea) following the manufacturer’s guidelines. The quantity and quality of the obtained RNA were assessed using NanoDrop equipment (Thermo Scientific, USA). According to the instructions, complementary DNA (cDNA) was synthesized utilizing the Mircury Lna Universal cDNA Synthesis Kit. qRT-PCR was carried out to detect the expression level of miR-383, *Bcl-2, Caspase-3, CD44*, and *MMP-2* using the BioFACT^TM^ 2X Master Mix (Korea) in the StepOnePlus Real-Time PCR System (Applied Biosystems, USA). The *GAPDH* gene served as an internal control for mRNA expression, while the *U6* gene functioned as the internal control for miRNA expression. Relative quantification was conducted using the 2^-ΔΔCt^ method, and each reaction was replicated independently three times.^26^ Primer pair sequences can be found in Table 1.

**Table 1:** Sequences of Primers Used in This Study

**Gene**	**Forward and Reverse**	**Sequences**
*GAPDH*	F	5ˊ-CAAGATCATCAGCAATGCCT-3ˊ
	R	5ˊ-GCCATCACGCCACAGTTTCC-3ˊ
*U6*	F	5ˊ-CTTCGGCAGCACATATACTAAAATTGG-3ˊ
	R	5ˊ-TCATCCTTGCGCAGGGG-3ˊ
*Bcl-2*	F	5ˊ-CTGTGGATGACTGAGTACCTG-3ˊ
	R	5ˊ-GAGACAGCCAGGAGAAATCA-3ˊ
*Caspase-3*	F	5ˊ-GGAAGCGAATCAATGGACTCTGG-3ˊ
	R	5ˊ-GCATCGACATCTGTACCAGACC-3ˊ
*MMP-2*	F	5ˊ-AGCGAGTGGATGCCGCCTTTAA-3ˊ
	R	5ˊ-CATTCCAGGCATCTGCGATGAG-3ˊ
*CD44*	F	5′-CCAGAAGGAACAGTGGTTTGGC-3′
	R	5′-ACTGTCCTCTGGGCTTGGTGTT-3′

###  Annexin V/ Propidium Iodide Apoptosis Assay and DAPI Staining

 The apoptosis induction rate was assessed using the Annexin V FITC apoptosis detection kit. Cells were divided into four groups: miR-383, cisplatin, combined miR-383/cisplatin, and a control group. The cells were collected, rinsed with PBS, and suspended in a 100 μL binding buffer. Subsequently, each sample was treated with 5 μL annexin-V and 5 μL propidium iodide (PI) and then incubated for 20 minutes in darkness. Apoptosis induction rates were measured via MACS Quant Flow Cytometry, and the FlowJo software was employed for data analysis. Additionally, to further explore the apoptosis induction rate in treated cells, DAPI staining was conducted. Briefly, A549 cells were cultured at a density of 10 × 10^3^ cells per well in a 96-well plate. Following treatment and incubation, the cells were washed with PBS, fixed with 4% paraformaldehyde for 2‒4 hours at 37°C, and then treated with Triton X-100 (0.1%) for 10 minutes. After washing, the cells were stained with DAPI for 10 minutes. The fragmented chromatin of apoptotic cells was observed using the Cytation 5 system (BioTek).^27^

###  Autophagy

 The simultaneous effect of miR-383 and cisplatin on autophagy activation was evaluated by applying monodansylcadaverine (MDC) staining. Briefly, in a six-well plate, A549 cells were seeded at a density of 2 × 10^5^. After treatment and incubation steps, the cells were washed with PBS, stained with 500 μl MDC and incubated at 37 °C for 10 minutes. Next, the cells were re-washed, harvested, and analyzed on flow cytometry. Eventually, the results were analyzed by the FlowJo software.^28^

###  Wound-Healing Assay 

 The migration of A549 cells was assessed through the wound-healing assay. After transfection with miR-383, the cells were plated in a 24-well plate and allowed to reach a confluence of 70–80%. Subsequently, a wound area was created within the cell monolayer using a sterile yellow pipette tip. The media of wells were exchanged and treated with cisplatin. For the wound-healing assay, cell migration was quantified by measuring the wound area at different time points (0, 12, 24, and 48 hours) using an inverted light microscope (Optika, Italy), ensuring reproducibility and accuracy in the analysis.^29^

###  Colony Formation Assay

 Cells subjected to treatment (1000 cells per well) were placed into a six-well plate and cultured for 7 days. Subsequently, the cells were immobilized using 4% paraformaldehyde, rinsed, and subjected to staining with a 0.05% crystal violet solution for 40 minutes. Finally, the colonies formed were counted for each group. The colonies were counted manually to ensure accuracy and consistency in the analysis.^30^

###  Statistical Analysis

 The experiments were conducted in triplicate, and the GraphPad Prism 8.0 Software was utilized for data analysis. Results are presented as means ± standard deviation (SD). Normality of data distribution was assessed using the Shapiro-Wilk test to determine the appropriateness of parametric tests. Statistical analysis involved using the Student’s *t*-test for normally distributed data when comparing two groups and one-way analysis of variance (ANOVA) for multiple groups, followed by Tukey’s post hoc test for multiple comparisons. For non-normally distributed data, non-parametric alternatives such as the Mann-Whitney U test and Kruskal-Wallis test with Dunn’s post hoc correction were applied. A significance level of *P* < 0.05 indicated statistical significance in the observed differences.

## Results

###  miRNA-383 Synergistically Enhanced Cisplatin-Induced Cytotoxicity

**Figure 1:**
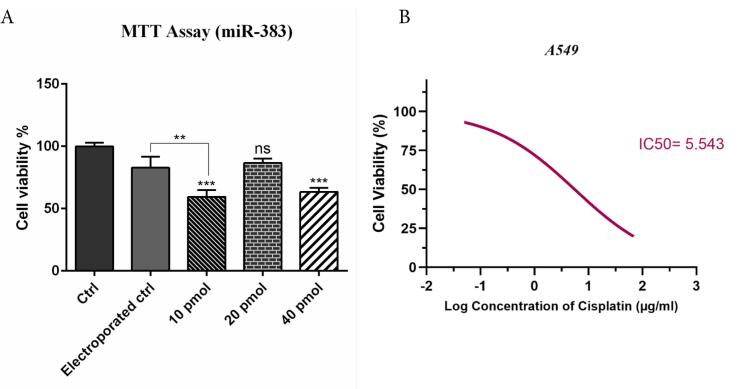
**A)** Effect of miR-383 Transfection on A549 Cell Viability. A549 cells were transfected with miR-383 at different concentrations (10, 20, and 40 pmol). The MTT assay was used to evaluate cell viability. A significant reduction in viability was observed at 10 pmol and 40 pmol concentrations (*P* < 0.001), while the 20 pmol group and the electroporation control (without miRNA) showed no significant difference (ns). **B)** Cisplatin Sensitivity and IC50 Reduction. The dose-response curve shows the cytotoxic effect of cisplatin alone versus cisplatin + miR-383 treatment. Co-treatment with miR-383 significantly enhanced cisplatin sensitivity, as shown by the leftward shift in the viability curve. The IC50 of cisplatin decreased from 28.91 μg/mL (cisplatin alone) to 2.319 μg/mL (cisplatin + miR-383), indicating a synergistic effect. (***P < 0.001, **P < 0.01, ns = non-significant)

 To evaluate whether miR-383 treatment could enhance the sensitivity of the A549 cells to cisplatin, the MTT assay was carried out. First, the optimum concentration of miR-383 transfection into cells was determined. A549 cells were transfected with different doses of miR-383 (10, 20, 40 pmol), and after 48 hours, cell viability was assessed via MTT assay. Results demonstrated that 10 and 40 pmol of miR-383 significantly decreased the survival of cells (*P* < 0.0001), so the 10 pmol dose was selected for transfection of A549 cells (Figure 1A). As illustrated in Figure 1B, cisplatin treatment could individually considerably reduce cell proliferation in a dose-dependent manner. Moreover, co-treatment of miR-383/cisplatin remarkably reduced the survival of cells compared to groups treated with cisplatin individually. In addition, results revealed that miR-383 transfection increased the sensitivity of A549 cells to cisplatin. As depicted in Figure 1B, the IC50 of cisplatin decreased significantly from 28.91 μg/mL in cells treated individually to 2.319 μg/mL in the combination group (*P* < 0.0001). The percentage decrease in IC50 value of cisplatin in the presence of miR-383 was approximately 91.98% (Formula 1).

**Formula 1.** Initial and final IC50 values: initial_ IC50 = 28.91 μg/mL, final_ IC50 = 2.319 μg/mL,

 Percentage decrease calculation

 Percentage_decrease = ((initial_ IC50 - final_ IC50) / initial_ IC50) * 100

###  Combined Effect of miR-383 and Cisplatin on Cell Apoptosis

**Figure 2:**
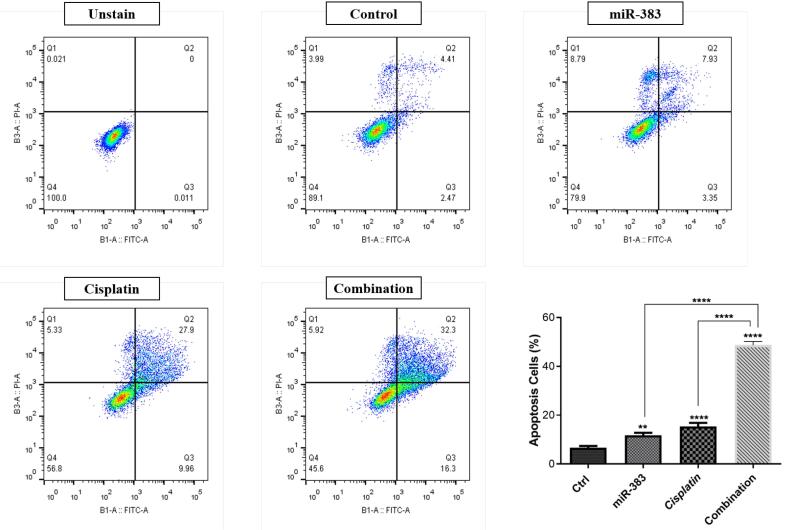
Rate of apoptosis induction in treatment groups. Flow cytometry analysis using Annexin V-FITC/PI staining shows that miR-383 and cisplatin independently induced apoptosis in A549 cells, increasing the apoptosis induction rate from 6.88% in control to 11.28% (*P* < 0.01) and 37.86% (*P* < 0.0001), respectively. Notably, the combination treatment of miR-383 and cisplatin significantly elevated apoptosis to 48.6% (*P* < 0.0001), indicating a synergistic effect. (****P < 0.0001, **P < 0.01).

**Figure 3:**
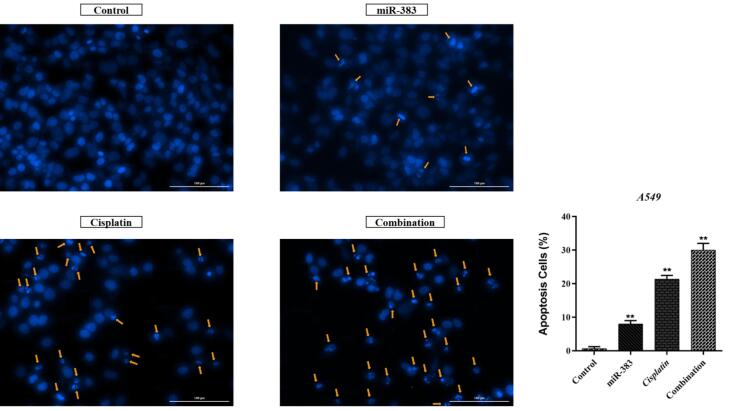
DNA fragmentation and apoptosis induction in miR-383/cisplatin-treated A549 cells. The combined treatment group shows the most pronounced DNA fragmentation, supporting the higher apoptosis rate observed in flow cytometry (**P < 0.01)

**Figure 4:**
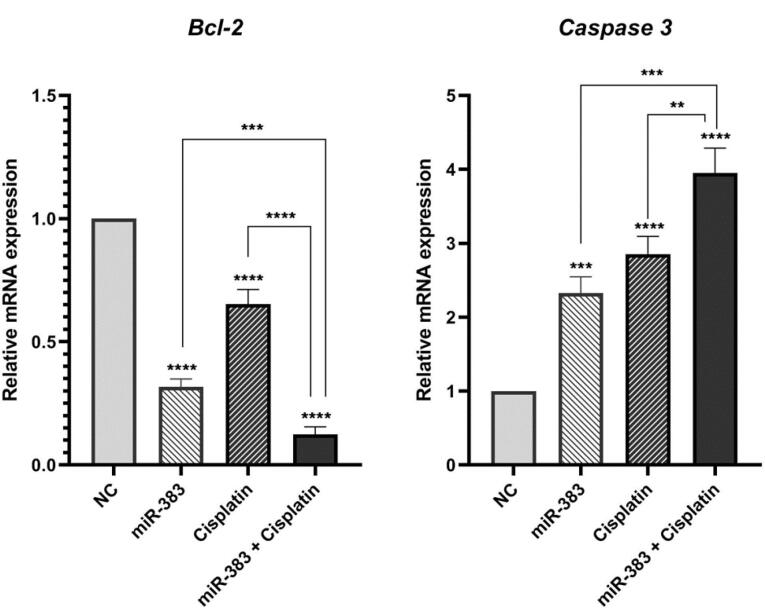
Assessment of *Bcl-2 *and* Caspase-3* Gene Expression Levels Using qRT-PCR. miR-383 and cisplatin reduced *Bcl-2* expression and increased *Caspase-3* levels. The combination treatment significantly enhanced these effects. (**** *P* < 0.0001, *** *P*< 0.001, **P < 0.01)

 The co-treatment effect of miR-383/cisplatin on apoptosis induction was measured by employing Annexin V/PI and DAPI staining. Results exhibited that miR-383 and cisplatin could individually significantly enhance the apoptosis induction in A549 cells from 6.88% to 11.28% (*P* < 0.01) and 37.86% (*P* < 0.0001), respectively. Also, data showed that the apoptosis rate in the combination group increased significantly to 48.6% compared to individual treatments (*P* < 0.0001) (Figure 2). DAPI staining revealed that co-treatment of miR-383/cisplatin simultaneously increased the apoptosis induction rate, further confirming apoptosis induction through treatment groups (Figure 3). Following this, qRT-PCR was utilized to examine the expression levels of genes involved in regulating apoptosis, aiming to elucidate the mechanisms behind apoptosis. Our findings revealed that both miR-383 and cisplatin, when administered separately, notably decreased the expression of *Bcl-2* (a pro-survival gene) compared to the control group (*P* < 0.0001). Also, in the combination group, *Bcl-2* expression was remarkably downregulated compared to singly treated groups (*P* < 0.0001). Furthermore, in miR-383 and cisplatin treated cells, a significant upregulation was observed for *Caspase-3* (as a pro-apoptotic gene) compared to control (*P* < 0.001 and *P* < 0.0001, respectively). In the combination group, the expression level of *Caspase-3* was remarkably upregulated compared to singly treated groups (*P* < 0.0001) (Figure 4).

###  miR-383 and Cisplatin Effect on Autophagy Induction

**Figure 5:**
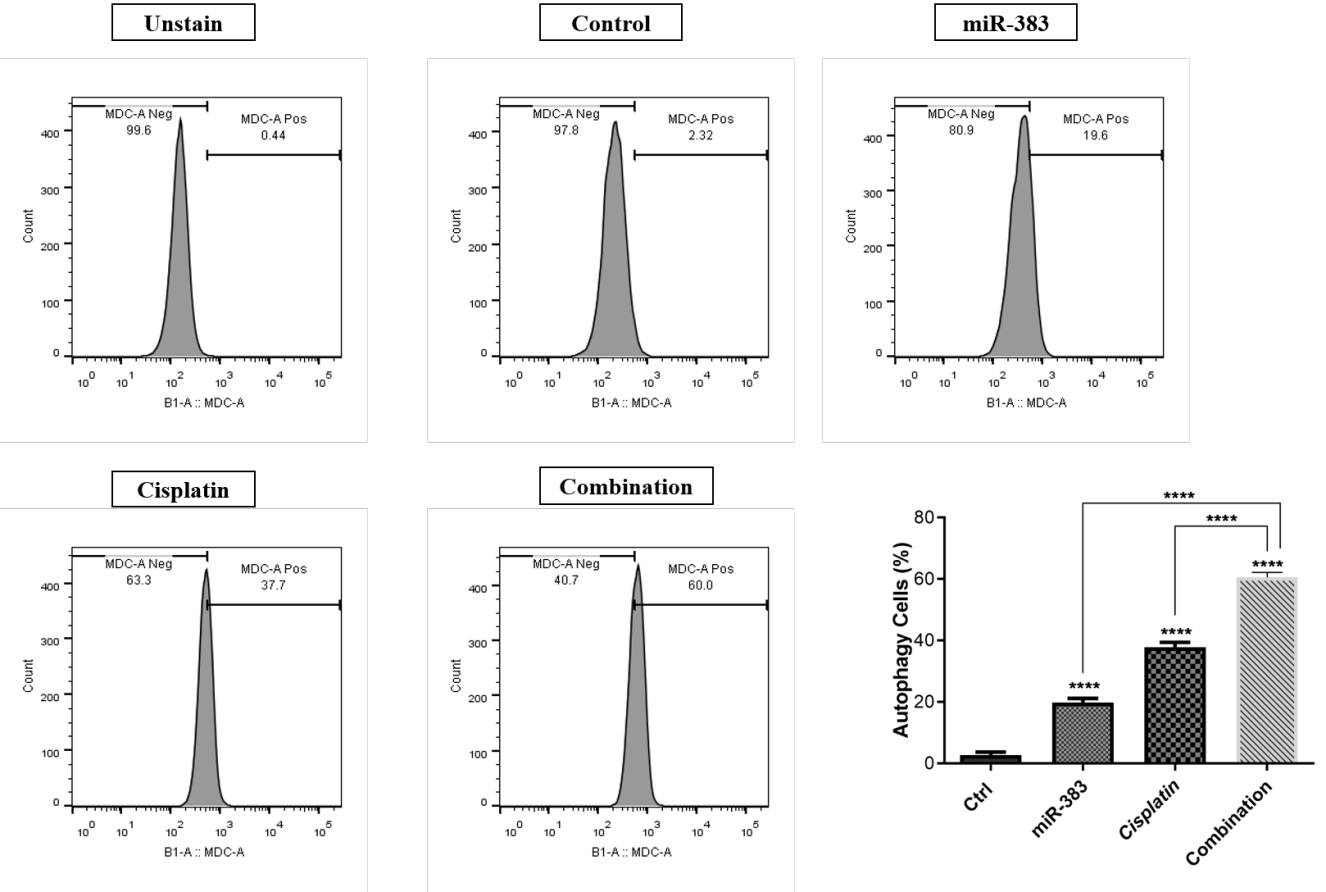
Rate of Autophagy Across Treatment Groups of A549 cells. miR-383 and cisplatin increased autophagy to 18.5% and 34%, respectively. Their combination further enhanced autophagy to 55.9%. (****P < 0.0001)

 Through flow cytometry analysis, we examined the impact of combined therapy on activating autophagy. Our findings demonstrated that individual treatments with miR-383 and cisplatin notably elevated the autophagy rate to 18.5% and 34%, respectively, compared to the control group’s rate of 1.07% (*P* < 0.0001). Moreover, the combination of miR-383/cisplatin could increase the autophagy rate (55.9%) more effectively than single treatment groups (Figure 5).

###  miR-383 and Cisplatin Inhibited A549 Cell Migration

**Figure 6:**
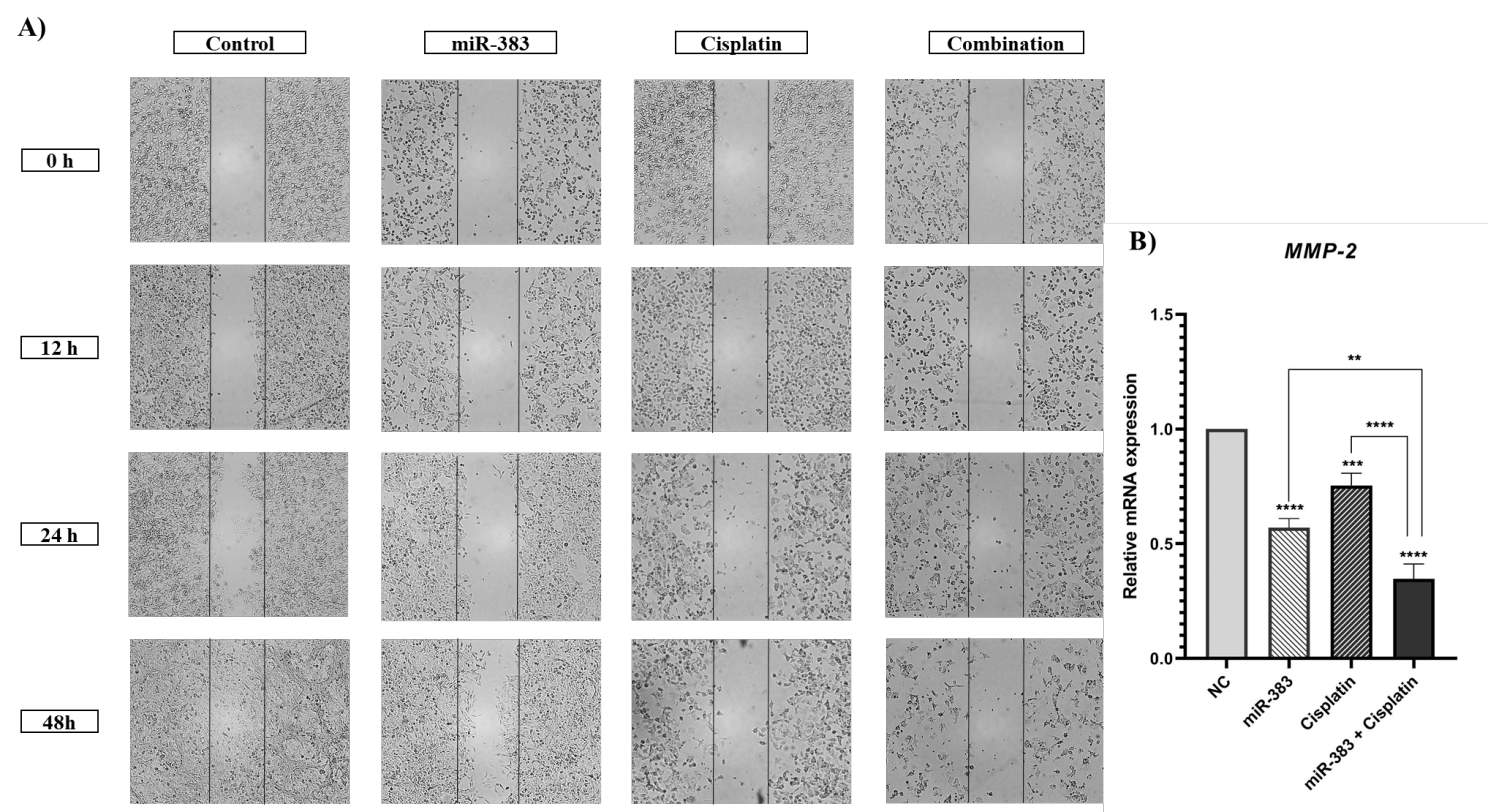
**A)** Effect of cisplatin and miR-383 on cell migration assessed by wound-healing assay and imaged at various times (0, 12, 24, and 48 h). The combination of miR-383 and cisplatin significantly inhibited cell migration. **B)** Expression level of *MMP-2 *assessed using qRT-PCR. The combination of miR-383 and cisplatin considerably downregulated *MMP-2* expression more effectively than individual treatments. (****P < 0.0001, ***P < 0.001, **P < 0.01)

**Figure 7:**
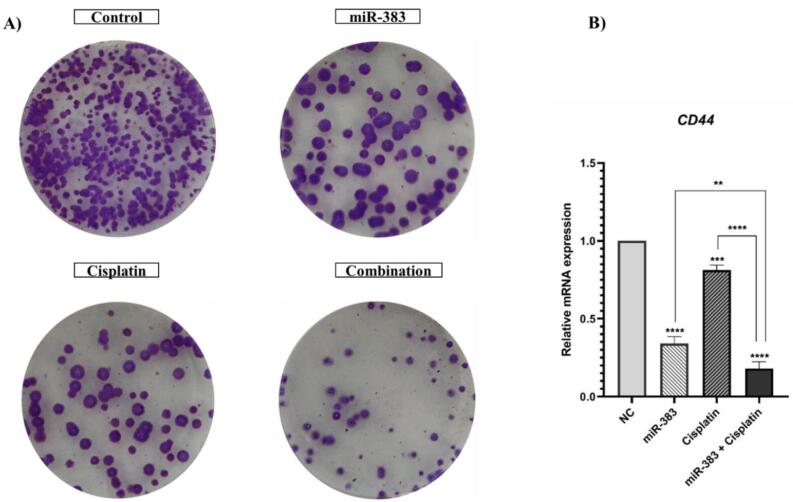
**A)** Separate and combined effect of cisplatin and miRNA-383 on the colony formation ability. miR-383 and cisplatin individually suppressed the colony formation ability of A549 cells compared to the control. The combination treatment showed a stronger inhibitory effect than either treatment alone. **B)** Expression levels of CD44 were measured employing qRT-PCR. miR-383 and cisplatin significantly downregulated CD44 expression. The combination treatment further suppressed CD44 expression more effectively than individual treatments. (****P < 0.0001, ***P < 0.001, **P < 0.01)

 The wound-healing assay was conducted to investigate the effect of combined treatment of cisplatin and miR-383 on cell migration. According to Figure 6, our results indicated a notable reduction in the migration of the miR-383 treated group compared to control cells. Additionally, cisplatin alone was able to decrease cell migration ability. More importantly, the results illustrated that combination therapy inhibited cell migration more effectively than single treatment groups. Subsequently, the expression level of *MMP-2*, as a metastasis-related gene, was evaluated using qRT-PCR. According to Figure 7, the results showed that cells treated individually with miR-383 or cisplatin could significantly downregulate *MMP-2* expression (*P* < 0.0001). The combination of miR-383 and cisplatin could considerably suppress the expression of *MMP-2* more effectively than the singly treated groups (*P* < 0.0001).

###  miRNA-383 and Cisplatin Treatment Suppressed the Colony Formation Ability of A549 Cells

 The effects of miRNA-383 and cisplatin treatment on cancer stemness properties of cells were assessed by utilizing the colony formation assay. Our results showed that the combined treatment of miRNA-383 and cisplatin significantly suppressed the colony formation ability of A549 cells (Figure 7A). Also, combined treatment obviously suppressed colony formation more effectively than singly treated groups. In addition, we used qRT‐PCR to evaluate the effects of miRNA-383 and cisplatin on CD44 gene expression (as a marker for cancer stem cells). Our results exhibited that cells treated with miR-383 and cisplatin separately could significantly downregulate CD44 expression, while combined treatment could remarkably suppress CD44 expression more than single treatment groups (*P* < 0.0001) (Figure 7B).

## Discussion

 Globally, LC is one of the leading causes of cancer-related deaths. However, the LC mortality rate is now decreasing due to advertisements, campaigns and declining tobacco consumption in the United States.^31^ NSCLC is a term that includes various types of LCs, most notably adenocarcinoma, large cell carcinoma, and squamous cell carcinoma (SCC). Adenocarcinoma is the most prevalent type of LC and includes 20% of all LC cases. Another type of NSCLC is SCC which usually originates from the tracheobronchial tree. Treatment strategy varies based on the functional status of patients, tumor stage, molecular characteristics of the disease, and comorbidities. In patients with stage I, II, or III NSCLC, treatment methods include surgery, chemotherapy, radiation therapy, or a combined modality approach.^32^ miRNA dysregulation can modulate various critical pathways, including the PI3K/AKT/mTOR and MAPK signaling pathways, which play key roles in regulating drug resistance mechanisms in cancer. These pathways influence cell survival, proliferation, and apoptosis, contributing to treatment failure.^33^ Drug resistance is considered one of the main reasons for the failure of chemotherapy. Dysregulation of miRNAs affects gene expression in drug resistance mechanisms, such as apoptosis, cell cycle control, and DNA damage repair. Altered expression of miRNAs can activate alternative compensatory pathways and/or modulate drug target protein expressions, leading to drug resistance. Many studies have shown miRNAs’ role in regulating the response to different drugs in various cancers like LC.^34^ miRNAs can regulate LC resistance by interacting with compounds related to drug resistance, signal pathways, and cell apoptosis, affecting cancer cells sensitivity to drugs. So, miRNAs can be applied as a specific target for LC therapy and play a crucial role in the early prognosis, diagnosis, and treatment of LC.^35^

 In the current study, we investigated the combination effect of miRNA-383 and cisplatin on LC cells. According to the results obtained from the MTT assay, pretreatment of A549 cells with miR-383 could significantly increase the chemosensitivity of cells to cisplatin treatment. Consequently, the combined use of miR-383 and cisplatin significantly reduced cancer cell proliferation and viability compared to cisplatin treatment alone. These outcomes align with previous research. Fang et al documented the down-regulation of miR-383 expression in hepatocellular cancer cells and highlighted that transfecting hepatocellular cancer cells with miR-383 could curb cell proliferation.^36^ Cui et al showed that in LoVo and HT-29 cells, upregulation of miR-383 reduced proliferation and decreased viability.^37^ The upregulation of miR-383 in ovarian cancer cells also suppressed proliferation and increased the chemosensitivity of cells to paclitaxel.^15^

 In addition, we illustrated that treatment of A549 cells with miR-383 and cisplatin alone considerably increased the rate of apoptosis compared to the control cells. Moreover, the findings indicated a notably higher level of apoptosis in the combination-treated group compared to cells subjected to individual treatments. For deeper exploration, the qRT-PCR technique was employed to gauge the expression of apoptosis-associated genes like *Bcl-2* and *Caspase-3*. BCL-2 proteins encompass a set of interconnected molecules distributed throughout the animal kingdom. In humans, the BCL-2 family proteins segregate into three categories: 1) pro-apoptotic effectors (BAK, BAX, BOK), 2) pro-apoptotic BH3-only proteins (BID, BIK, BIM, BAD, HRK), and 3) anti-apoptotic proteins (BCL-XL, BCL-2, BCL-W, BFL-1/A1). The pro-apoptotic BCL-2 effectors facilitate apoptosis by permeabilizing the mitochondrial outer membrane (MOMP). Conversely, anti-apoptotic BCL-2 proteins, including BCL-2 itself, impede apoptosis by restraining MOMP.^38,39^ Caspases, proteases crucial in orchestrating cellular demise, participate significantly in signaling cascades culminating in cell death. They segregate into inflammatory caspases (caspase-1, -4, -5, -11), initiator caspases (caspase-2, -8, -9, -10), and executioner caspases (caspase-3, -6, -7). Impairment in the caspase function stands as a pivotal factor contributing to tumor development.^40,41^ According to the results, combined use of miR-383 and cisplatin on A549 cells showed remarkable upregulation of the *Caspase-3* and downregulation of *Bcl-2* compared to individual groups. Consistent with our results, Wan et al investigated the synergistic effect of cisplatin and paeonol on esophageal cancer cell lines. Their results indicated that treatment of cells by cisplatin could significantly increase the expression level of *Caspase-3* and decrease the expression of *Bcl-2.*^42^

 Li et al indicated that transfection of gastric cancer cells with miR-383 suppressed proliferation and induced apoptosis. In addition, overexpression of miR-383 significantly decreased *Bcl-2* expression in cancer cells.^43^ Moreover, miR-383 could inhibit cell growth and induce cell apoptosis in hepatocellular carcinoma cells. Also, miR-383 increased the expression of *Caspase-3*, *-9*, and *Bax* and decreased the expression of *Bcl-2* in these cells.^10^

 Autophagy, a highly conserved cellular degradation mechanism, crucially sustains cellular equilibrium by sequestering dysfunctional organelles, aggregated proteins, and pathogens and routing them to lysosomes for subsequent degradation.^44^ Autophagy exhibits a multifaceted impact on cancer, fostering tumor cell survival by providing recycled metabolites to fuel growth and regulating mitochondrial function via mitophagy. Cancer therapy can induce the autophagy mechanism that contributes to cancer cell survival.^45^ Considering its importance, we investigated the role of miR-383 and cisplatin in the induction of autophagy in A549 cells. Our results revealed that treating A549 cells with miR-383 and cisplatin separately could induce autophagy compared to control cells. Moreover, the concurrent administration of miR-383 with cisplatin resulted in increased induction of autophagy compared to single treatment groups. Previously, Xu et alreported that miR-383 increased apoptosis, autophagy, and reactive oxygen species production in human glioma U87 cells.^13^

 Cell migration is a complex process essential for physiological development, regeneration, and tissue repair. However, cell migration promotes metastasis, the leading cause of mortality in cancer patients.^46^ Cancer stem cells, which can renew themselves, play a crucial role in starting tumors, increasing resistance to cancer treatments, and promoting the spread of cancer to other parts of the body. Transcriptomic analyses in various cancers have revealed a strong connection between immune patterns and stem cell characteristics, indicating possible biological connections between key aspects of cancer.^47^ In our study, the wound-healing and colony formation assays were performed to further explore the effects of cisplatin and miR-383 on A549 cells migration capability and stemness features. The obtained results indicated that the combination therapy could remarkably inhibit migration capability and diminish the number of colonies and colony formation ability compared to cells treated with a single agent. Moreover, the expression levels of *MMP-2* and *CD44* genes involved in metastasis pathways and stemness features were assessed. The results showed that in cells treated with the combination of cisplatin and miR-383, the downregulation of *MMP-2* and *CD44 *genes was considerably greater than in singly treated groups. Matrix metalloproteinases (MMPs) are crucial enzymes engaged in numerous cellular functions. They are recognized for their capacity to break down the extracellular matrix and aid cell movement outside the cell. MMPs play roles in diverse mechanisms like cell growth, specialization, and blood vessel formation, as well as cell death. While they serve important functions in processes like healing wounds and repairing muscle tissue, MMPs can also contribute to various health issues such as inflammatory diseases, artery hardening, heart attacks, and cancer progression.^48^ CD44 is a non-kinase transmembrane glycoprotein widely involved in several cancers as a cancer stem cell marker. Cells overexpressing CD44 have several cancer stem cell features, such as epithelial-mesenchymal transition capability, self-renewal, and resistance to radiotherapy and chemotherapy.^49^ Consistent with our results, a recent study by Yang et al on the synergistic effect of sorafenib and cisplatin in human osteosarcoma cells showed that cisplatin could significantly suppress cell proliferation, colony formation, migration, and invasion.^50^ In another study, the combination of Liquiritigenin and cisplatin was investigated in the invasion and metastasis of melanoma cells. The results demonstrated that cisplatin could remarkably reduce cell migration and invasion. Moreover, cisplatin decreased the expression of MMP-2/9, PI3K, and p-AKT.^51^ The transfection of breast cancer cells with miR-383 also could suppress proliferation and migration and promote apoptosis by inhibiting PD-L1. Furthermore, the results indicated that miR-383 may exert its tumor-suppressive effect by inhibiting the PI3K/AKT/mTOR pathway.^52^ Studies have proven that miR-383 is downregulated in prostate cancer, essential in determining tumor initiation potential and affecting metastasis by directly regulating CD44. Moreover, low expression of miR-383 in clinical prostate cancer tissues was linked to a negative prognosis.^53^ This compelling evidence highlights miRNAs as potential therapeutic targets that could enhance chemotherapy responses and improve drug sensitivity in tumor cells, offering promising prospects for cancer treatment.

## Conclusion

 In summary, our study highlights the significant therapeutic potential of combining cisplatin and miR-383 for treating LC, a combination that has not been previously reported in this context. While the effectiveness of this combination has been observed in other cancers, its application to LC represents a novel approach. The combination treatment effectively reduced the proliferation of A549 and induced cell apoptosis by regulating the expression of *Bcl-2* and *Caspase-3*. Additionally, this treatment promoted autophagy induction. Furthermore, it successfully inhibited cell migration and stemness potential by reducing the expression levels of the *MMP-2* and *CD44* genes. This study’s limitations include using a single cell line, lack of *in-vivo* validation, and failure to assess gene expression at the protein level. Investigating related signaling pathways would provide further mechanistic insights. These limitations need to be addressed in future research to ensure the effectiveness of this approach. These findings suggest that the combination of cisplatin and miR-383 may enhance therapeutic efficacy in LC; however, further studies are needed to confirm its clinical potential.
